# Diseases of the Skin

**Published:** 1877-11

**Authors:** 


*A Practical Treatise on Diseases of the Skin.* By Louis A. Duhring, M. D., etc. Philadelphia: J. B. Lippincott & Co., 1877. Buffalo: Theo. H. Butler.

Dr. Duhring is well known as a writer on Dermatology, and the two numbers issued of his Atlas of Skin Diseases have met with such general favor that a Treatise on the subject from his pen will be looked upon with interest by all familiar with the author's reputation. The author has aimed to produce a work which shall be of a practical character, which shall be adapted to the general practitioner, who is, we are sorry to confess, lamentably ignorant of the subject. Dr. Duhring we are glad to see, has not yielded to the temptation, which seem to assail almost all writers upon skin diseases, to present a classification of his own. While the best classification has doubtless not been yet made, and will no doubt be undiscovered for some time to come, the habit which almost every new writer has of presenting one of his own invention tends only to delay the hoped for perfect one, by making "confusion more confounded." The nomenclature and classification, with some slight modifications of Hebra, have been adopted, and for that reason if for no other the work will favorably impress most dermatologists as Hebras' teachings seem to predominate in the large medical centers, and the work will therefore not clash to any considerable extent with views already held as to arrangement. We cannot at this time enter into a general review of Dr. Duhring's work, nor can we indeed give it the notice which its merits deserve. We hope in connection with future numbers of the "Atlas" to be able to say something of the author's general views. The book is well and sufficiently illustrated, some of the drawings by Dr. Van Harlingen are excellently made.

The author's style is concise and clear, and he manages to make what is frequently in most books but dry detail, at once interesting and instructive. The treatment advised is such as evidently has met the test of practical experience, and is judiciously applied. Taken together with the Atlas when finished, it will take a leading position among Text books of Skin Diseases.

